# How to Write and Publish a Research Paper for a Peer-Reviewed Journal

**DOI:** 10.1007/s13187-020-01751-z

**Published:** 2020-04-30

**Authors:** Clara Busse, Ella August

**Affiliations:** 1 Department of Maternal and Child Health, University of North Carolina Gillings School of Global Public Health, 135 Dauer Dr, 27599 Chapel Hill, NC USA; 2 Department of Epidemiology, University of Michigan School of Public Health, 1415 Washington Heights, Ann Arbor, MI 48109-2029 USA

**Keywords:** Manuscripts, Publishing, Scientific writing

## Abstract

Communicating research findings is an essential step in the research process. Often, peer-reviewed journals are the forum for such communication, yet many researchers are never taught how to write a publishable scientific paper. In this article, we explain the basic structure of a scientific paper and describe the information that should be included in each section. We also identify common pitfalls for each section and recommend strategies to avoid them. Further, we give advice about target journal selection and authorship. In the online resource 1, we provide an example of a high-quality scientific paper, with annotations identifying the elements we describe in this article.

**Electronic supplementary material:**

The online version of this article (10.1007/s13187-020-01751-z) contains supplementary material, which is available to authorized users.

## Introduction

Writing a scientific paper is an important component of the research process, yet researchers often receive little formal training in scientific writing. This is especially true in low-resource settings. In this article, we explain why choosing a target journal is important, give advice about authorship, provide a basic structure for writing each section of a scientific paper, and describe common pitfalls and recommendations for each section. In the online resource 1, we also include an annotated journal article that identifies the key elements and writing approaches that we detail here. Before you begin your research, make sure you have ethical clearance from all relevant ethical review boards.

### Select a Target Journal Early in the Writing Process

We recommend that you select a “target journal” early in the writing process; a “target journal” is the journal to which you plan to submit your paper. Each journal has a set of core readers and you should tailor your writing to this readership. For example, if you plan to submit a manuscript about vaping during pregnancy to a pregnancy-focused journal, you will need to explain what vaping is because readers of this journal may not have a background in this topic. However, if you were to submit that same article to a tobacco journal, you would not need to provide as much background information about vaping.

Information about a journal’s core readership can be found on its website, usually in a section called “About this journal” or something similar. For example, the Journal of Cancer Education presents such information on the “Aims and Scope” page of its website, which can be found here: https://www.springer.com/journal/13187/aims-and-scope.

Peer reviewer guidelines from your target journal are an additional resource that can help you tailor your writing to the journal and provide additional advice about crafting an effective article [1]. These are not always available, but it is worth a quick web search to find out.

### Identify Author Roles Early in the Process

Early in the writing process, identify authors, determine the order of authors, and discuss the responsibilities of each author. Standard author responsibilities have been identified by The International Committee of Medical Journal Editors (ICMJE) [2]. To set clear expectations about each team member’s responsibilities and prevent errors in communication, we also suggest outlining more detailed roles, such as who will draft each section of the manuscript, write the abstract, submit the paper electronically, serve as corresponding author, and write the cover letter. It is best to formalize this agreement in writing after discussing it, circulating the document to the author team for approval. We suggest creating a title page on which all authors are listed in the agreed-upon order. It may be necessary to adjust authorship roles and order during the development of the paper. If a new author order is agreed upon, be sure to update the title page in the manuscript draft.

In the case where multiple papers will result from a single study, authors should discuss who will author each paper. Additionally, authors should agree on a deadline for each paper and the lead author should take responsibility for producing an initial draft by this deadline.

## Structure of the Introduction Section

**Fig. 1:**
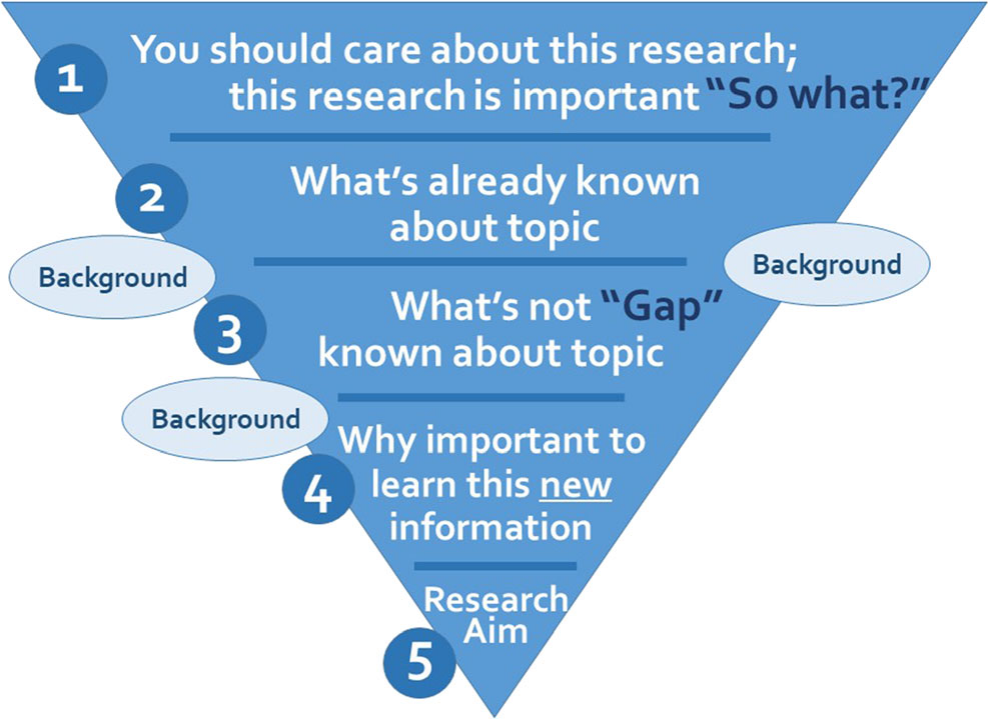
The main elements of the introduction section of an original research article. Often, the elements overlap

The introduction section should be approximately three to five paragraphs in length. Look at examples from your target journal to decide the appropriate length. This section should include the elements shown in Fig. 1. Begin with a general context, narrowing to the specific focus of the paper. Include five main elements: why your research is important, what is already known about the topic, the “gap” or what is not yet known about the topic, why it is important to learn the new information that your research adds, and the specific research aim(s) that your paper addresses. Your research aim should address the gap you identified. Be sure to add enough background information to enable readers to understand your study. Table 1 provides common introduction section pitfalls and recommendations for addressing them.

**Table 1:** Common introduction section pitfalls and recommendations

Pitfall	Recommendation
Introduction is too generic, not written to specific readers of a designated journal.	*Choose a target journal and write to its readers.* Visit your target journal’s website and investigate the journal’s readership. If you are writing for a journal with a more general readership, like PLOS ONE, you should include more background information. A narrower journal, like the Journal of the American Mosquito Control Association, may require less background information because most of its readers have expertise in the subject matter.
Citations are inadequate to support claims.	*Cite all statements that could be challenged.* If a claim could be debated, it should be supported by one or more citations. To find articles relevant to your research, consider using open-access journals, which are available for anyone to read for free. A list of open-access journals can be found here: https://guides.lib.umich.edu/c.php?g=283428&p=1884017. You can also find open-access articles using PubMed Central: https://www.ncbi.nlm.nih.gov/pmc/
The research aim is vague.	*Include enough key information to allow readers to imagine the analysis.* Be sure that your research aim contains essential details like the setting, population/sample, study design, timing, dependent variable, and independent variables. Using such details, the reader should be able to imagine the analysis you have conducted.

## Methods Section

The purpose of the methods section is twofold: to explain how the study was done in enough detail to enable its replication and to provide enough contextual detail to enable readers to understand and interpret the results. In general, the essential elements of a methods section are the following: a description of the setting and participants, the study design and timing, the recruitment and sampling, the data collection process, the dataset, the dependent and independent variables, the covariates, the analytic approach for each research objective, and the ethical approval. The hallmark of an exemplary methods section is the justification of why each method was used. Table 2 provides common methods section pitfalls and recommendations for addressing them.

**Table 2:** Common methods section pitfalls and recommendations

Pitfall	Recommendation
The author only describes methods for one study aim, or part of an aim.	*Make sure the methods are complete.* Be sure to check that the methods describe all aspects of the study reported in the manuscript.
There is not enough (or any) justification for the methods used.	*Justify each approach and variable.* You must justify your choice of methods because it greatly impacts the interpretation of results. State the methods you used and then defend those decisions. For example, justify why you chose to include the measurements, covariates, and statistical approaches.

## Results Section

The focus of the results section should be associations, or lack thereof, rather than statistical tests. Two considerations should guide your writing here. First, the results should present answers to each part of the research aim. Second, return to the methods section to ensure that the analysis and variables for each result have been explained.

Begin the results section by describing the number of participants in the final sample and details such as the number who were approached to participate, the proportion who were eligible and who enrolled, and the number of participants who dropped out. The next part of the results should describe the participant characteristics. After that, you may organize your results by the aim or by putting the most exciting results first. Do not forget to report your non-significant associations. These are still findings.

Tables and figures capture the reader’s attention and efficiently communicate your main findings [3]. Each table and figure should have a clear message and should complement, rather than repeat, the text. Tables and figures should communicate all salient details necessary for a reader to understand the findings without consulting the text. Include information on comparisons and tests, as well as information about the sample and timing of the study in the title, legend, or in a footnote. Note that figures are often more visually interesting than tables, so if it is feasible to make a figure, make a figure. To avoid confusing the reader, either avoid abbreviations in tables and figures, or define them in a footnote. Note that there should not be citations in the results section and you should not interpret results here. Table 3 provides common results section pitfalls and recommendations for addressing them.

**Table 3:** Common results section pitfalls and recommendations

Pitfall	Recommendation
The text focuses on statistical tests rather than associations.	*Focus on associations instead of statistical tests.* The relationships between independent and dependent variables are at the heart of scientific studies and statistical tests are a set of strategies used to elucidate such relationships. For example, instead of reporting that “the odds ratio is 3.4,” report that “women with exposure X were 3.4 times more likely to have disease Y.” There are several ways to express such associations, but all successful approaches focus on the relationships between the variables.
Causal words like “cause” and “impact” are used inappropriately	Only some study designs and analytic approaches enable researchers to make causal claims. Before you use the word “cause,” consider whether this is justified given your design. Words like “associated” or “related” may be more appropriate.
The direction of association unclear.	*Be explicit about direction of association.* Instead of “X is associated with Y,” say “an increase in variable X is associated with a decrease in variable Y,” a sentence which more fully describes the relationship between the two variables.

## Discussion Section

**Fig. 2:**
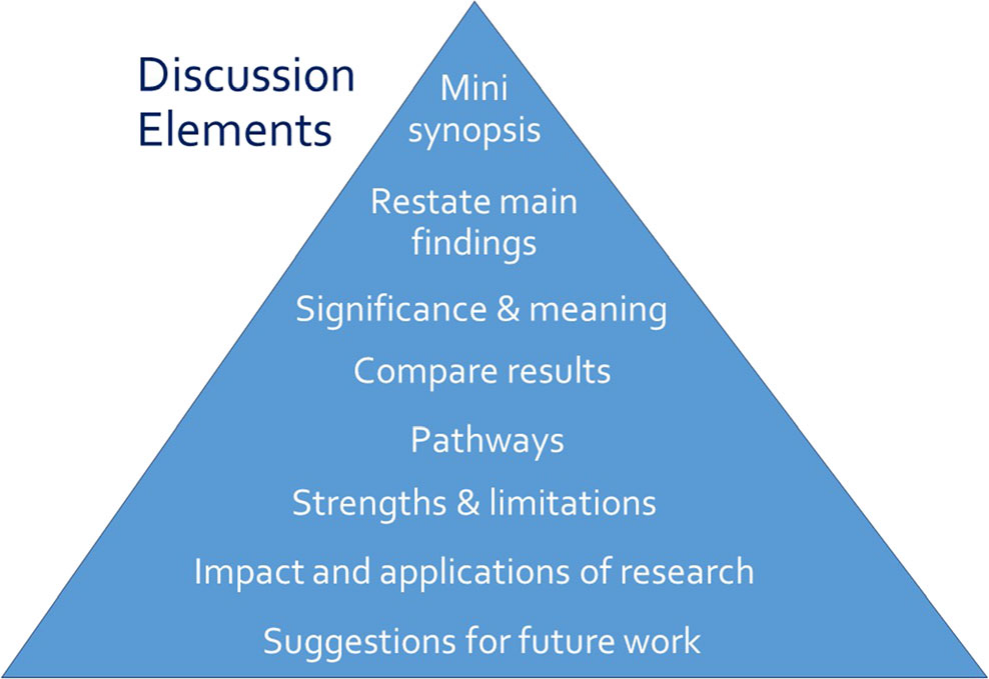
Major elements of the discussion section of an original research article. Often, the elements overlap

Opposite the introduction section, the discussion should take the form of a right-side-up triangle beginning with interpretation of your results and moving to general implications (Fig. 2). This section typically begins with a restatement of the main findings, which can usually be accomplished with a few carefully-crafted sentences.

Next, interpret the meaning or explain the significance of your results, lifting the reader’s gaze from the study’s specific findings to more general applications. Then, compare these study findings with other research. Are these findings in agreement or disagreement with those from other studies? Does this study impart additional nuance to well-accepted theories? Situate your findings within the broader context of scientific literature, then explain the pathways or mechanisms that might give rise to, or explain, the results.

Journals vary in their approach to strengths and limitations sections: some are embedded paragraphs within the discussion section, while some mandate separate section headings. Keep in mind that every study has strengths and limitations. Candidly reporting yours helps readers to correctly interpret your research findings.

The next element of the discussion is a summary of the potential impacts and applications of the research. Should these results be used to optimally design an intervention? Does the work have implications for clinical protocols or public policy? These considerations will help the reader to further grasp the possible impacts of the presented work.

Finally, the discussion should conclude with specific suggestions for future work. Here, you have an opportunity to illuminate specific gaps in the literature that compel further study. Avoid the phrase “future research is necessary” because the recommendation is too general to be helpful to readers. Instead, provide substantive and specific recommendations for future studies. Table 4 provides common discussion section pitfalls and recommendations for addressing them.

**Table 4:** Common discussion section pitfalls and recommendations

Pitfall	Recommendation
The author repeats detailed results or presents new results in the discussion section.	*Focus on how your study fits into the scientific literature.* Recall from Fig. 1 that the discussion section should take the shape of a triangle as it moves from a specific restatement of the main findings to a broader discussion of the scientific literature and implications of the study. Specific values should not be repeated in the discussion. It is also not appropriate to include new results in the discussion section.
The author fails to describe the implication of the study’s limitations.	*Where possible, identify the implications of your study’s limitations.* No matter how well-conducted and thoughtful, all studies have limitations. Candidly describe how the limitations affect the application of the findings.
Statements about future research are too generic.	*Be specific!* Is the relationship between exposure and outcome not well-described in a population that is severely impacted? Or might there be another variable that modifies the relationship between exposure and outcome? This is your opportunity to suggest areas requiring further study in your field, steering scientific inquiry toward the most meaningful questions.

## Follow the Journal’s Author Guidelines

After you select a target journal, identify the journal’s author guidelines to guide the formatting of your manuscript and references. Author guidelines will often (but not always) include instructions for titles, cover letters, and other components of a manuscript submission. Read the guidelines carefully. If you do not follow the guidelines, your article will be sent back to you.

Finally, do not submit your paper to more than one journal at a time. Even if this is not explicitly stated in the author guidelines of your target journal, it is considered inappropriate and unprofessional.

### Title

Your title should invite readers to continue reading beyond the first page [4, 5]. It should be informative and interesting. Consider describing the independent and dependent variables, the population and setting, the study design, the timing, and even the main result in your title. Because the focus of the paper can change as you write and revise, we recommend you wait until you have finished writing your paper before composing the title.

Be sure that the title is useful for potential readers searching for your topic. The keywords you select should complement those in your title to maximize the likelihood that a researcher will find your paper through a database search. Avoid using abbreviations in your title unless they are very well known, such as SNP, because it is more likely that someone will use a complete word rather than an abbreviation as a search term to help readers find your paper.

## Summary

**Fig. 3:**
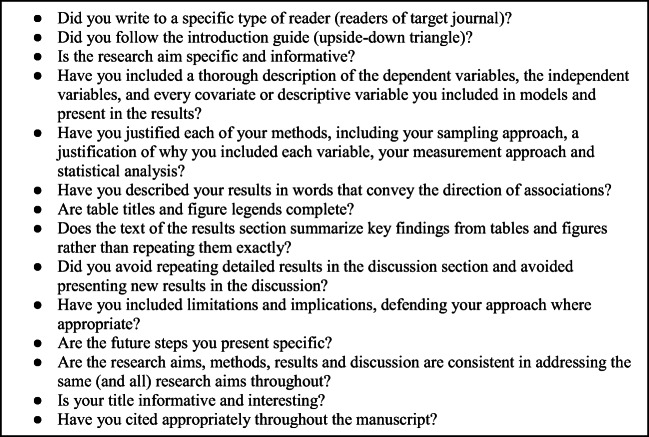
Checklist for manuscript quality

After you have written a complete draft, use the checklist (Fig. 3) below to guide your revisions and editing. Additional resources are available on writing the abstract and citing references [5]. When you feel that your work is ready, ask a trusted colleague or two to read the work and provide informal feedback. The box below provides a checklist that summarizes the key points offered in this article.

## Electronic supplementary material

ESM 1 (PDF 362 kb)

## Data Availability

Blinded.
