# Supplementary material for: How to Write and Publish a Research Paper for a Peer-Reviewed Journal
Source: J Cancer Educ. 2020 Apr 30;36(5):909–13. doi: 10.1007/s13187-020-01751-z (PMC8520870; doi:10.1007/s13187-020-01751-z)
Supplement: Supplementary file 1 — (PDF 362 kb) [file 13187_2020_1751_MOESM1_ESM.pdf]

original report

Acceptability of Human Papillomavirus Self-Sampling for Cervical Cancer Screening in an Indigenous Community in Guatemala

The title of this descriptive paper highlights the main dependent variable, setting and population.

abstract

Purpose Cervical cancer rates in Latin America are higher than those in developed countries, likely because of the lower prevalence of screening. Specifically, less than 40% of women in Guatemala are regularly screened and even fewer women are screened in indigenous communities. Current screening strategies—Pap smears and visual inspection with acetic acid—might not be the most effective methods for controlling cancer in these settings. We thus investigated the potential of self-collection of cervical samples with testing for human papillomavirus (HPV) to help prevent cervical cancer in an indigenous community in Guatemala.

Patients and Methods A community representative random sample of 202 indigenous women age 18 to 60 years residing in Santiago Atitlan, Guatemala, were surveyed to assess knowledge of and risk factors for HPV and cervical cancer. Women were then invited to self-collect a cervical sample using HerSwab collection kits to assess the prevalence of HPV and the acceptability of self-sampling.

Results Of 202 women who completed the survey, 178 (89%) provided a self-sample. In all, 79% of these women found the test comfortable, 91% found the test easy to use, and 100% reported they were willing to perform the test periodically as a screening method. Thirty-one samples (17%) were positive for at least one of 13 high-risk HPV types, and eight (4.5%) were positive for HPV 16/18.

Conclusion HPV testing by using self-collected samples was well accepted, suggesting that it is a plausible modality for cervical cancer screening in indigenous communities. Further studies are needed to assess rates of follow-up after a positive test and to determine whether these findings extend to other indigenous and nonindigenous communities in Guatemala and Latin America.

J Glob Oncol 3. © 2017 by American Society of Clinical Oncology Licensed under the Creative Commons Attribution 4.0 License

Anna Gottschlich

Alvaro Rivera-Andrade

Edwin Grajeda

Christian Alvarez

Carlos Mendoza Montano

Rafael Meza

Author affiliations and support information (if applicable) appear at the end of this article.

Corresponding author: Rafael Meza, PhD, University of Michigan, School of Public Health II, Room 5533, 1415 Washington Heights, Ann Arbor, MI 48109-2029; Twitter: @meza_rafa; e-mail: rmeza@umich.edu.

Why this research is important

INTRODUCTION

Cervical cancer (CC) is preventable with appropriate screening and treatment. Pap smears, the most common form of screening, allow physicians to detect and manage pre-cancerous lesions before they develop into CC.[1]

What is already known about this topic

Because of the success of screening programs that use the Pap smear, CC rates are low in most high-income countries.[2,3] Nonetheless, CC is the third most common cancer worldwide and a leading cause of death among women in low- and middle-income countries (LMICs).[4] Unfortunately, Pap smears are infrequently used in LMICs because they are expensive and require physicians, pathologists, and cytotechnicians to perform the procedure and interpret the results.[3,5] Even in LMICs with screening programs, rates of participation tend to be low[6] because Pap smears must be collected and analyzed at hospitals or other high-resource health facilities that women may not have access to.

Background information

In addition, if women have abnormal results, they must return for follow-up assessment and/or treatment, which creates greater time and financial burdens.[7] The logistics of sample collection by health care providers, which then must be sent to laboratories, tested, and returned, can also be challenging in these settings. There are also cultural barriers that preclude the use of screening methods associated with sexually transmitted diseases (STDs).

Hence, many LMICs have adopted CC screening programs that use visual inspection with acetic acid (VIA). VIA involves placing acetic acid on the cervix and looking for a change in color to

Background information

Why readers should care about this research

© 2017 by American Society of Clinical Oncology Licensed under the Creative Commons Attribution 4.0 License

Downloaded from ascopubs.org by 50.197.99.173 on January 31, 2020 from 050.197.099.173
Copyright © 2020 American Society of Clinical Oncology. All rights reserved.

Background information

detect lesions. This procedure is less costly and invasive than Pap smears and can be performed by trained laypersons in low-resource health facilities.[7-9] In addition, VIAs give the option to treat women with cervical lesions immediately. Thus VIA is often called a "see/screen-and-treat" or "one-visit" approach.[7] Previous studies have shown that VIA screening helps reduce CC incidence and mortality in low-resource settings.[8] However, VIA shares some of the same barriers associated with Pap smears, so despite these efforts, CC incidence and mortality remain high in many LMICs, presumably because of persistent low rates of screening with either approach.

Human papillomavirus (HPV) infections are responsible for more than 90% of CC cases.[10,11] There are 13 types of high-risk HPV associated with development of CC.[12] Of these, types 16 and 18 account for approximately 70% of all cases.[13] Cervical HPV tests have high sensitivity (approximately 90%) and specificity (> 80%).[14,15] Women who test positive for high-risk HPV should follow up with a Pap smear and/or VIA or treatment, depending on each country's setting and resources,[16] but a negative test means the risk of developing CC in the next few years is minimal, lower than the risk after a negative Pap smear.[17] Furthermore, when Pap smears are performed only on women who have tested positive for HPV, the relatively low sensitivity of screening by using Pap smears is significantly improved.[15,18] Thus, primary screening for high-risk HPV before referral for Pap smear or VIA has been proposed as an alternative CC screening method. Unfortunately, HPV testing is expensive and requires infrastructure not readily available in many LMICs. Nonetheless, research is underway to develop low-cost HPV tests that can be used with minor infrastructure requirements.[19-23]

What is already known about this topic

Self-collection HPV kits have been developed to allow women to collect their own cervicovaginal samples at home and send these to a testing facility through the mail or by other means. Studies in several countries have compared the accuracy of HPV self-collection samples with samples obtained by a physician and have assessed the acceptability of self-collection in different populations.[5,24-30] Some studies have provided women with self-collection kits, but at medical facilities before collection by a physician rather than at the woman's home. In these studies, self-collection has been shown to have sensitivity similar to that of physician-collected samples,[5,24-28] and self-collection has been found to be highly acceptable in many settings.[24,26-28,31] This suggests that self-collection could be helpful to increase CC screening rates in LMICs, once cost- and infrastructure-efficient HPV tests have been developed.

What is not known: the gap in the literature

However, few studies have provided participants with the opportunity to try these in community settings outside of medical facilities; thus, it is not clear whether they would be an accepted form of primary CC screening.

Why it's important to learn the information identified in the gap

Guatemala has one of the highest levels of CC morbidity and mortality in the region. Age-standardized annual incidence and mortality rates are 22.3 and 12.5 per 100,000 women, respectively,[11] largely because less than 40% of Guatemalan women (who have a relatively high prevalence of HPV[32-34]) have ever been screened for CC.[6,35]

What is known about this topic

There have been self-collection studies conducted in Latin America, a region in which CC morbidity and mortality are particularly high,[5,36-39]

What is not known about this topic

although few have tested the acceptability of HPV self-collection in community rather than clinical settings.

Why it is important to learn the new information the study provides

Moreover, HPV self-collection has not been studied in indigenous populations in Latin America, who tend to have less access to health facilities and higher levels of stigma associated with physician-administered vaginal and STD tests.[40] Thus, it is important to assess the acceptability of HPV self-collection kits and tests and determine the potential of HPV testing as a screening modality in these settings.[37]

Study aims

We thus conducted a cross-sectional study in an indigenous population in Lake Atitlan, Guatemala, to assess knowledge of HPV and CC, provide women with the opportunity to collect a self-sample in their home and report their feelings and experiences, and assess HPV prevalence in indigenous populations.

PATIENTS AND METHODS

Description of study design and setting, and overview of data collection approach

We conducted a cross-sectional study in Santiago Atitlan, an indigenous community of 45,000 residents in Guatemala. Data were collected by using electronic surveys and self-collection kits.

Study Population

Description of study population

This community is almost exclusively Tz'utujil, a Mayan indigenous group.

Description of sampling and recruitment strategy

We sampled 212 women age 18 to 60 years from nine neighborhoods that encompass 85% of the population of Santiago Atitlan. Population data were obtained from the local municipality. We followed a stratified sampling approach by first allocating samples of size N_c to each neighborhood according to its relative population size $(c = 1, \ldots, 9)$ and then randomly selecting a sample of N_c blocks. One house was randomly selected per block, in which one woman was interviewed.

Downloaded from ascopubs.org by 50.197.99.173 on January 31, 2020 from 050.197.099.173
Copyright © 2020 American Society of Clinical Oncology. All rights reserved.

Table 1. General Population Characteristics (N = 202)

Characteristic	No.	%	Mean	SD
Age, years			34.5	8.8
Education				
No formal	80	39.6		
Primary	85	42.1		
More than primary	37	18.32		
Daily income (Q)			32.8	19.8
Literate				
Yes	123	60.9		
No	79	39.1		
Married/united	173	85.6		
Age at marriage, years			19.2	3.7
Use health services	181	89.6		
Breast examination	10	5.0		
Mammogram	7	3.5		
Pap smear	134	66.3		
Time since last Pap smear (n = 134)				
< 6 months	16	11.9		
6 months to 1 year	8	6.0		
1-5 years	81	60.4		
> 5 years	29	21.6		
VIA	18	8.9		
Smoker	1	0.5		
Use alcohol	28	13.9		
Use Depo-Provera	109	54.0		
Use birth control pill	40	19.8		
Use IUD	6	3.0		
Use condom				
Always	3	1.5		
Almost always	5	2.5		
Sometimes	10	5.0		
Rarely	8	4.0		
Never	164	81.2		
Unknown	12	5.9		
No. of pregnancies			3.2	2.5
No. of children			2.9	1.9
Age at first pregnancy, years			20.3	4.0
Family member with cervical cancer	13	6.4		
Age at first sexual relation, years			19.1	3.9
No. of lifetime partners			1.2	0.5
Knowledge of HPV	30	14.9		
Diagnosed with cervical cancer	0			
Urban	165	81.7		

(Continued on following page)

If more than one woman in a house was eligible, the woman who had the next upcoming birthday was selected. Only women ages 25 to 54 years were eligible to provide a self-collected sample for HPV testing because women outside these ages are not eligible to receive screening using Pap smears or VIA according to Guatemalan CC screening guidelines. Menstruating and pregnant women were also excluded from self-collection. We chose to interview women outside the screening range because, although the focus of the study was on acceptability, we were interested in learning about the health practices and risk factors for all adult women.

Description of sampling and recruitment strategy

Inclusion and exclusion criteria

Justification of inclusion and exclusion criteria for interviews

Survey

The survey component was designed by using the Qualtrics application. It included 143 questions about demographics, preventive health care practices, and HPV and CC knowledge and risk factors. The survey also assessed the acceptability of and feelings toward HPV self-collection. Questions were developed by using the STEPwise Approach to Surveillance (STEPS) survey, The University of North Carolina's Family Health Study Survey, and the University of Michigan's Michigan HPV and Oropharyngeal Cancer study.[35,41] Four trained community health workers (CHWs) fluent in Tz'utujil and Spanish conducted the surveys.

Description and citation of data collection instrument

Information on the dependent variables and descriptive variables

The survey was written in English and then translated to Spanish by native speakers from the study team. The survey was piloted in Guatemala City and in households in Santiago Atitlan. After each pilot, surveyor notes were reviewed and appropriate revisions were made. At the end of each day, surveys were uploaded to the server, ensuring that the participant's data could no longer be accessed, except by members of the study team.

Notice that the procedural details here are very specific

HPV Self-Collected Samples

We used Eve Medical HerSwab self-collection HPV kits. Each kit came with an instructions card written in Spanish with step-by-step infographs explaining the collection process. The CHWs were trained on the procedure and on how to explain the instructions to the participants in their native language.

Upon interview completion, each eligible participant was asked about her interest in collecting a sample for HPV testing. If the participant agreed, the CHWs explained the instructions, and the participant collected a sample in a private room in the household. The collection kit comprised a plastic handle and brush. The woman inserted the brush into her vagina and then turned a crank on the handle to extend the brush. The woman then removed the brush and cranked it back by using

Data collection is described in enough detail to enable replication of the study

Downloaded from ascopubs.org by 50.197.99.173 on January 31, 2020 from 050.197.099.173
Copyright © 2020 American Society of Clinical Oncology. All rights reserved.

Table 1. General Population Characteristics (N = 202) (Continued)

Characteristic	No.	%	Mean	SD
Severity of CC				
Not	2	1.0		
A little	3	1.5		
Moderate	69	34.2		
Very	35	17.3		
Extremely	93	46.0		

Abbreviations: CC, cervical cancer; HPV, human papillomavirus; IUD, intrauterine device; Q, quetzal; VIA, visual inspection with acetic acid.

the handle. She then returned the kit to the CHWs. Afterward, each participant completed a five-question survey assessing the level of ease and comfort associated with the collection and her willingness to self-collect periodically as a form of CC screening. Finally, CHWs encouraged participants to attend free VIA screening clinics at their local public hospital.

Information on the laboratory analysis of specimens

Samples were sent to an independent, nonprofit laboratory in Guatemala City (Asociación de Salud Integral) for testing and were tested by using the Anyplex II[42] HPV-28 kit, which tests for 13 high-risk HPV types according to the International Agency for Research on Cancer classification,[12] as well as 15 low-risk types (Data Supplement).

Notice the justification of the decision not to collect contact information

To ensure the privacy and confidentiality of the participant's information, given the sensitivity of the survey questions and the HPV test, no contact information was collected in this pilot study; thus, participants could not be contacted by the study team with their results. Instead, participants were told to call for their results 10 days after collection by using an identification number. Announcements were made daily on the local radio for 1 month after the end of recruitment that reminded women to call for their results. Participants were informed only if they tested positive for one of the 13 high-risk types.

Statistical Analyses

Description of statistical analysis for each study aim

Post–self-collection survey questions were analyzed to determine the acceptability of HPV self-collection as a form of CC screening. Two additional outcomes were analyzed: positive HPV results and previous Pap smear or VIA results. Crude comparisons between these and relevant covariates were run by using log-binomial regression, and then models were run adjusting for other covariates. Statistical analyses were conducted by using SAS software Version 9.4 (SAS Institute, Cary, NC).

Human Subjects Approval

Statement of ethical board approval

The University of Michigan Institutional Review Board approved study protocols (HUM00096559). All participants gave oral, informed consent before participation. The consent was documented by signature from one of the CHWs.

RESULTS

Information on the final sample of participants included in the study and their characteristics

Of 481 women who were asked to participate through door-to-door recruitment, 212 women enrolled (44% acceptance rate), with 202 (95%) completing the survey. Ten women chose to withdraw, and their data were destroyed. Participants' mean age was 34.5 years, and more than 80% had primary education at most (Table 1). One hundred thirty-five women (67%) reported previous CC screening with Pap smears and/or VIA (Table 1). Women with previous Pap smear and/or VIA testing tended to be older, married, and with a higher number of children and pregnancies, suggesting that access to screening is strongly tied to reproductive care. Whereas only 31 participants (15%) reported previous knowledge of HPV, 188 (93%) were interested in and willing to collect a self-sample for HPV testing (Table 2). Of these, 178 (88%) were eligible and provided a sample.

Table 2. Acceptability of Self-Collection Tests (N = 202)

Variable	No.	%
Knowledge about HPV	30	14.9
Previous Pap smear/VIA	135	66.8
Intent to self-collect	188	93.1
Urban (n = 165)	153	92.7
Rural (n = 37)	35	94.6
Self-collected sample	178	88.1
Prefer home screening	163	80.7
Prefer self-screening	162	80.2
No. of participants who performed the test	178	88.1
Comfort of test		
Comfortable	140	78.7
Neutral	13	7.3
Uncomfortable	25	14.0
Ease of test		
Easy	162	91.0
Neutral	3	1.7
Difficult	13	7.3
Willingness to redo the test	178	100
Called for test results	163	91.6

Abbreviations: HPV, human papillomavirus; VIA, visual inspection with acetic acid.

Downloaded from ascopubs.org by 50.197.99.173 on January 31, 2020 from 050.197.099.173
Copyright © 2020 American Society of Clinical Oncology. All rights reserved.

Table 3. HPV Distribution (N = 178)

Status	No.	%
HPV positive	37	20.8
High-risk HPV positive	31	17.4
Positive for types 16/18	8	4.5

Abbreviation: HPV, human papillomavirus.

Table abbreviations are clearly defined

Notice that the results section is divided into sub-sections, making it easy to scan and navigate

Self-Collection Acceptability

Of these 178 women, 79% found the kit comfortable to use, and 91% found it easy to use. Upon collection, 100% reported that they were willing to use the test periodically as a form of CC screening, and more than 80% said they preferred to screen themselves at home rather than with a physician in a doctor's office (Tables 2 and 3). Because identifying information was not collected, the study team was unable to actively return results; however, more than 90% of participants called to receive their own results.

HPV Prevalence

Thirty-seven (21%) of 178 women tested positive for one of 28 types of HPV, and 31 (17%) tested positive for a high-risk type (Table 3). HPV 16 had the highest prevalence with seven women testing positive, followed by HPV 53 and 56 (six women tested positive for each), and HPV 59 (five women tested positive). Of the four strains with the highest prevalence, all except HPV 53 are high risk. Figure 1 shows the HPV type distribution in the study population.

HPV Infection

The number of lifetime sexual partners was significantly higher in women who tested positive for HPV. Characteristics comparing women by HPV test results can be found in Table 4; characteristics comparing women by their number of sexual partners can be found in the Data Supplement. Exposure covariates in the final model include current age, level of education, age at first pregnancy, and age at first sexual encounter. Other covariates were also explored, including age at marriage and other demographic factors.

After adjustment, the association became statistically nonsignificant, but it did show a prevalence ratio (PR) greater than 1 (crude PR, 2.18; 95% CI, 1.07 to 4.43; *P* = .03; adjusted PR, 1.42; 95% CI, 0.68 to 2.97; *P* = .34; regression tables are provided in the Data Supplement).

Previous Screening

The use of health services was statistically significantly higher in women who had a previous Pap smear or VIA. Characteristics of women with and without a history of screening are presented in Table 5, and characteristics of women categorized by use of health services are presented in the Data Supplement. The final adjusted model included age and education level, as well as the HPV test results. The participants' use of alcohol, as well as other demographic factors, were considered but not included in the final model.

The direction of each association is indicated

After adjustment, the association between use of health services and having had a previous Pap smear or VIA remained greater than 1 but was no longer significant (crude PR, 2.49; 95% CI, 1.26 to 4.93; *P* = .009; adjusted PR, 1.24; 95% CI, 0.93 to 1.66; *P* = .15; regression tables are provided in the Data Supplement).

DISCUSSION

We assessed the acceptability of HPV self-collection as an alternative to screening by using Pap smears or VIA in an indigenous community in Latin America. We found that self-collection kits had high acceptability and were preferred to physician screenings; a majority of women found the test kit comfortable and easy to use. We found a 17.4% prevalence of high-risk HPV, which is consistent with previous studies reporting a

Restatement of the study aim

Restatement of the study's main findings

Comparison of this study's findings with others in the literature

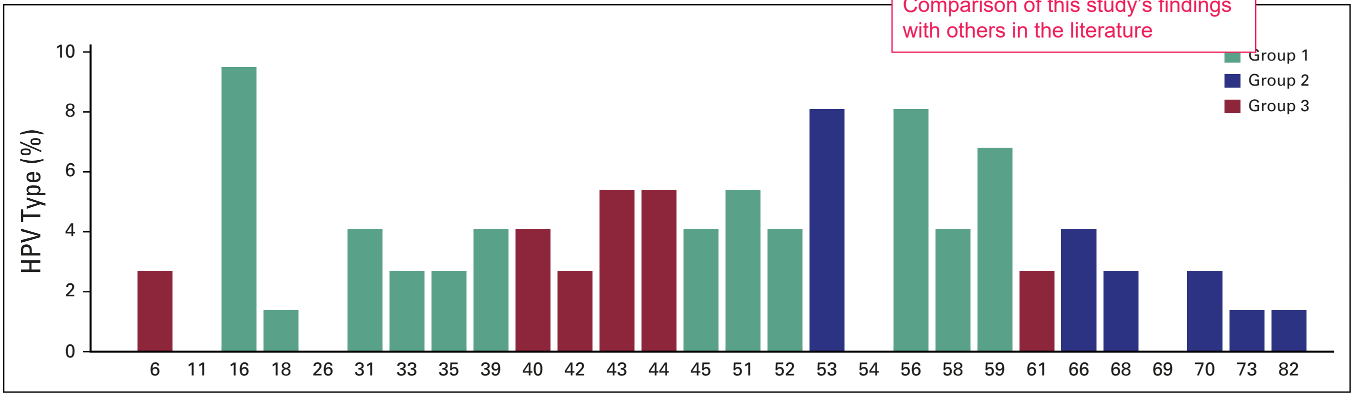

Fig 1. Distribution of human papillomavirus (HPV) genotypes among the 37 HPV-positive samples. Several women had prevalent infections with more than one type; thus, these percentages represent the number of infections from a specific type of HPV divided by the total number of HPV infections. Group 1: most potent HPV type known to cause cancer at several sites or sufficient evidence for cervical cancer; group 2: limited evidence in humans with varying levels of evidence for cervical cancer; group 3: no evidence in humans for cancer.

Downloaded from ascopubs.org by 50.197.99.173 on January 31, 2020 from 050.197.099.173
Copyright © 2020 American Society of Clinical Oncology. All rights reserved.

Table 4. Characteristics of HR HPV-Positive and HPV-Negative Participants in Guatemala (N = 178)

Characteristic	HR HPV-Positive				HR HPV-Negative				
	No.	%	Mean	SD	No.	%	Mean	SD	*P*
No. of patients	31				147				
Age, years*			35.4	9.6			34.3	8.7	.57
Education†									.2425
No formal	12	38.7			60	40.8			
Primary	17	54.8			62	42.2			
More than primary	2	6.5			25	17.0			
Pap/VIA†	26	83.9			101	68.7			.0897
Daily income (Q)*			33.1	19.0			33.2	20.8	.97
Lifetime sexual partners*			1.3	0.5			1.1	0.4	.04
Age at marriage, years*			17.4	2.9			19.4	3.6	.0041
Literate†	18	58.1			87	59.2			.9083
Married/united†	26	83.9			134	91.2			.2215
Use health services†	31	100			130	88.4			.0461
Use alcohol†	2	6.5			19	12.9			.3099
Use Depo-Provera	21	67.7			83	56.5			.2469
Use birth control pill†	7	22.6			32	21.8			.92
Use IUD†	2	3.2			4	2.7			.28
Use condom†	3	9.6			17	11.6			.76
No. of pregnancies*			4.0	2.7			3.1	2.4	.0662
No. of children*			3.1	1.9			2.8	1.9	.3769
Age at first pregnancy, years*			18.8	3.1			20.6	4.2	.0231
Age at first sexual relation, years*			17.2	3.0			19.1	3.5	.0027
Knowledge of HPV†	5	16.1			22	15.0			.7897

NOTE. *P* values at < .05 significance level.
Abbreviations: HPV, human papillomavirus; HR, high risk; IUD, intrauterine device; Q, quetzal; SD, standard deviation; VIA, visual inspection with acetic acid.
*P value obtained by using independent t test.
†P value obtained by using χ^2 test.

16.1% prevalence for Latin America.[43] We also investigated risk factors for HPV infection and previous Pap smears or VIAs, associations that became statistically nonsignificant after adjustment for other covariates. This could be the result of inconsistencies with self-reporting or perhaps because their partner's sexual history (which was not assessed) might be a stronger determinant of HPV risk in this community.

Discussion of possible reasons for this study's findings

This study was intended to serve as a first step to determine the potential of HPV screening in indigenous populations and also to provide baseline data for future longitudinal studies assessing the efficacy of HPV testing versus other screening modalities. Perhaps the most relevant finding is the high acceptability of self-collection and the willingness of the participants to engage in the study. In fact, 95% of participants completed the survey, 93% were interested in collecting self-samples, and more than 90% called to receive their results, numbers higher than expected. The study was very well received in the community, with strong support from local and health authorities, suggesting the potential to eventually implement HPV screening programs in this and other similar settings.

Practical applications of the study's findings

Implications of the study's results

Strengths of the study include the multiclustered community design, which allowed us to obtain a representative sample of the population, provided an opportunity for participants to try self-collection in their homes rather than at a clinic, and allowed local CHWs to perform recruitment and interviews. Because of the latter, interviews were conducted in the participants' native language, potentially making them more comfortable answering sensitive questions. In addition, data were collected electronically,

Notice that the authors begin this paragraph with the word "strengths," making it easy to locate the study's strengths

Downloaded from ascopubs.org by 50.197.99.173 on January 31, 2020 from 050.197.099.173
Copyright © 2020 American Society of Clinical Oncology. All rights reserved.

Table 5. Characteristics of Participants Who Had or Had Not Received Previous Screening (N = 202)

Characteristic	Previous Pap/VIA No.	Previous Pap/VIA %	Previous Pap/VIA Mean	Previous Pap/VIA SD	No Previous Pap/VIA No.	No Previous Pap/VIA %	No Previous Pap/VIA Mean	No Previous Pap/VIA SD	*P*
No. of participants	135				67				
Age, years*			36.3	8.3			30.9	8.8	< .001
Education†									.0486
No formal	53	39.3			27	40.3			
Primary	63	46.7			22	32.7			
More than primary	19	14.1			18	26.9			
HR HPV positive†	26	20.47			5	9.8			.0897
Daily income (Q)*			33.6	19.9			31.2	19.5	.43
Lifetime sexual partners*			1.2	0.5			1.2	0.4	.61
Age at marriage, years*			18.8	3.4			20.1	4.3	.05
Literate†	80	59.3			43	64.2			.4999
Married/united†	132	91.9			51	76.1			.0065
Use health services†	129	95.6			52	77.6			< .001
Frequency of health visits†									.142
Once a month or more	29	21.5			12	17.9			
Every 3-6 months	51	37.8			18	26.9			
Once a year or less	55	40.8			37	55.2			
Time since last visit to health services, years†									.208
< 1	98	72.5			42	62.7			
1-5	23	17.1			12	17.9			
> 5	7	5.2			4	6.0			
Never	7	5.2			9	13.4			
Breast examination†	9	6.7			1	1.5			.17
Mammogram†	7	5.2			0	0			.10
Use alcohol†	11	8.2			17	25.4			< .001
Use Depo-Provera†	92	68.2			17	25.4			< .001
Use birth control pill†	32	23.7			8	11.9			.0482
Use condom†	18	13.3			8	12			.78
No. of pregnancies*			3.7	2.5			2.1	4.3	< .001
No. of children*			3.1	2.0			2.4	1.5	.0138
Age at first pregnancy, years*			20.1	4.1			21.0	3.8	.1878
Family member with CC†	8	5.9			5	7.5			.6951
Age at first sexual relation, years*			18.6	3.3			20.2	4.8	.0201
Knowledge of HPV†	23	17.0			7	10.5			.2150

NOTE. *P* values at < .05 significance level.

Abbreviations: CC, cervical cancer; HPV, human papillomavirus; HR, high risk; Q, quetzal; SD, standard deviation; VIA, visual inspection with acetic acid.

**P* value obtained by using independent *t* test.

†*P* value obtained by using χ^2 test.

which eliminated the risk of errors from manual data entry. However, there are also important limitations. Given the cross-sectional design, participants might have misreported their history of screening and other risk factors, especially if there had been community educational programs or interventions that suggested that women should be screened for CC. Women may not have accurately remembered whether they had previously had a Pap smear or VIA (recall bias) or may not even be aware of whether

The study's limitations

Notice that the limitations are specific

Downloaded from ascopubs.org by 50.197.99.173 on January 31, 2020 from 050.197.099.173
Copyright © 2020 American Society of Clinical Oncology. All rights reserved.

Notice that the authors describe how they plan to address limitations in their future research

these procedures had been performed on them. Another limitation is that we were unable to assess whether HPV-positive women followed up on their results. This is the topic of our current work in multiple communities in Guatemala with a new study population that will be followed up after 6 months and 1 year.

Authors describe how future research should address the study limitations

In addition, this community has been exposed to prior health interventions and studies from multiple institutions.[44-46] Although these studies did not specifically discuss HPV and CC, the exposure to health interventions could be reflected in the women's knowledge of health issues and their willingness to try self-collection. In the future, it will be important to assess the acceptability of these tests in other indigenous communities with less exposure to studies and interventions.

Comparison of findings with other studies

Statement of this study's importance

The study results are consistent with those of previous studies conducted in Asia and Africa on the acceptability of self-screening for HPV.[25,27,29] However, to the best of our knowledge, this is the first study to assess self-collection in indigenous populations in Latin America. This is also one of the first studies to provide an opportunity for participants to collect a sample in a community setting rather than simply sharing their feelings toward self-collection or collecting at a clinic.

Discussion of this study's generalizability

This work assessed the acceptability of HPV self-screening in one community in Guatemala. Guatemala is a country with 23 languages and even more distinct communities, so our findings cannot be generalized to the whole population. It will be important to attempt to replicate the study in other parts of Guatemala and Latin America. Although it does seem that HPV self-collection screening could be a useful alternative to Pap smear or VIAs in these settings, this information alone does not allow us to make any determinations about whether this method of screening will reduce CC rates in developing countries. Women who tested positive for HPV should follow up with a doctor to receive Pap smears or VIAs or treatment. Hence, a logical next step would be to conduct longitudinal studies that compare rates of follow-up care among women who have tested positive with rates for those who have not been screened for HPV, as well as head-to-head comparisons between HPV-based versus Pap smear and VIA screening programs.[47] It is also important to continue developing new affordable and easy-to-use tests that could readily be implemented in low-income settings.[20-22]

Suggestions for future research are specific

The Ministry of Health in Guatemala is in the process of refining the National Cervical Cancer Prevention and Control program.[48] Following Pan-American Health Organization and WHO guidelines, the ministry has compiled a list of screening programs, some including HPV testing, that could be adopted. It will be the responsibility of each province (department) to determine which program best fits their needs and resources. We hope that our study, along with future evidence,[49] will help local and regional authorities identify the best CC screening alternative for their own settings.

Thorough discussion of how the study's findings might be applied

DOI: https://doi.org/10.1200/JGO.2016.005629
Published online on jgo.org on January 18, 2017.

AUTHOR CONTRIBUTIONS

Conception and design: Anna Gottschlich, Alvaro Rivera-Andrade, Christian Alvarez, Carlos Mendoza Montano, Rafael Meza

Collection and assembly of data: Anna Gottschlich, Alvaro Rivera-Andrade, Edwin Grajeda

Data analysis and interpretation: Anna Gottschlich, Rafael Meza

Manuscript writing: All authors

Final approval of manuscript: All authors

Accountable for all aspects of the work: All authors

AUTHORS' DISCLOSURES OF POTENTIAL CONFLICTS OF INTEREST

The following represents disclosure information provided by authors of this manuscript. All relationships are considered compensated. Relationships are self-held unless noted. I = Immediate Family Member, Inst = My Institution. Relationships may not relate to the subject matter of this manuscript. For more information about ASCO's conflict of interest policy, please refer to www.asco.org/rwc or ascopubs.org/jco/site/ifc.

Anna Gottschlich
No relationship to disclose

Alvaro Rivera-Andrade
No relationship to disclose

Edwin Grajeda
No relationship to disclose

Christian Alvarez
No relationship to disclose

Carlos Mendoza Montano
No relationship to disclose

Rafael Meza
No relationship to disclose

ACKNOWLEDGMENT

We thank the community health workers from Santiago Atitlan who performed the recruitment and interviews.

Downloaded from ascopubs.org by 50.197.99.173 on January 31, 2020 from 050.197.099.173
Copyright © 2020 American Society of Clinical Oncology. All rights reserved.

Affiliations

Anna Gottschlich, **Christian Alvarez**, and **Rafael Meza**, University of Michigan, Ann Arbor, MI; **Alvaro Rivera-Andrade** and **Carlos Mendoza Montano**, Institute of Nutrition of Central America and Panama; **Carlos Mendoza Montano**, Universidad Mariano Galvez de Guatemala; **Edwin Grajeda**, Universidad Rafael Landivar, Guatemala City, Guatemala; and **Alvaro Rivera-Andrade**, The Hebrew University, Jerusalem, Israel.

Support

Supported by the University of Michigan School of Public Health, Office of Global Health, by Grant No. U01CA182915 from the National Institutes of Health, National Cancer Institute training grant T32CA083654 (A.G.), and by the University of Michigan MCubed program.

Prior Presentation

Presented at The 2nd Annual Henry Ford Global Health Symposium, Detroit, MI, October 16, 2015; and the American Association for Cancer Research Annual Meeting, New Orleans, LA, April 16-20, 2016.

REFERENCES

1. Mayo Clinic: Tests and procedures: Pap smear—Definition. 2015 http://www.mayoclinic.org/tests-procedures/pap-smear/basics/definition/prc-20013038
2. International Agency for Research on Cancer: GLOBOCAN 2012: Estimated Cancer Incidence, Mortality and Prevalence Worldwide in 2012—Population Fact Sheets. http://globocan.iarc.fr/Pages/fact_sheets_population.aspx
3. Sankaranarayanan R, Budukh AM, Rajkumar R: Effective screening programmes for cervical cancer in low- and middle-income developing countries. Bull World Health Organ 79:954-962, 2001
4. Sherris J, Herdman C, Elias C: Cervical cancer in the developing world. West J Med 175:231-233, 2001
5. Boggan JC, Walmer DK, Henderson G, et al: Vaginal self-sampling for human papillomavirus infection as a primary cervical cancer screening tool in a Haitian population. Sex Transm Dis 42:655-659, 2015
6. American Cancer Society: Global Cancer Facts & Figures: 2nd Edition. 2008. http://oralcancerfoundation.org/wp-content/uploads/2016/03/acspc-027766.pdf
7. Ditzian LR, David-West G, Maza M, et al: Cervical cancer screening in low- and middle-income countries. Mt Sinai J Med 78:319-326, 2011
8. Nahar KN, Nessa A, Shamim S, et al: Role of VIA in cervical cancer screening in low-resource countries. Mymensingh Med J 20:528-535, 2011
9. African Population and Health Research Center, International Agency for Research on Cancer, World Health Organization: Prevention of cervical cancer through screening using visual inspection with acetic acid (VIA) and treatment with cryotherapy: A demonstration project in six African countries—Malawi, Madagascar, Nigeria, Uganda, the United Republic of Tanzania, and Zambia. 2012. http://www.who.int/reproductivehealth/publications/cancers/9789241503860/en/
10. Walboomers JM, Jacobs MV, Manos MM, et al: Human papillomavirus is a necessary cause of invasive cervical cancer worldwide. J Pathol 189:12-19, 1999
11. Luciani S, Cabanes A, Prieto-Lara E, et al: Cervical and female breast cancers in the Americas: Current situation and opportunities for action. Bull World Health Organ 91:640-649, 2013
12. Bouvard V, Baan R, Straif K, et al: A review of human carcinogens–Part B: Biological agents. Lancet Oncol 10:321-322, 2009
13. Bosch FX, Manos MM, Muñoz N, et al: Prevalence of human papillomavirus in cervical cancer: A worldwide perspective—International Biological Study on Cervical Cancer (IBSCC) Study Group. J Natl Cancer Inst 87:796-802, 1995
14. Benevolo M, Vocaturo A, Caraceni D, et al: Sensitivity, specificity, and clinical value of human papillomavirus (HPV) E6/E7 mRNA assay as a triage test for cervical cytology and HPV DNA test. J Clin Microbiol 49:2643-2650, 2011
15. de Kok IM, van Rosmalen J, Dillner J, et al: Primary screening for human papillomavirus compared with cytology screening for cervical cancer in European settings: Cost effectiveness analysis based on a Dutch microsimulation model. BMJ 344:e670, 2012
16. World Health Organization: WHO guidelines for screening and treatment of precancerous lesions for cervical cancer prevention. 2013. http://apps.who.int/iris/bitstream/10665/94830/1/9789241548694_eng.pdf
17. Gage JC, Schiffman M, Katki HA, et al: Reassurance against future risk of precancer and cancer conferred by a negative human papillomavirus test. J Natl Cancer Inst 106:dju251, 2014
18. Cuzick J, Arbyn M, Sankaranarayanan R, et al: Overview of human papillomavirus-based and other novel options for cervical cancer screening in developed and developing countries. Vaccine 26:K29-K41, 2008

Downloaded from ascopubs.org by 50.197.99.173 on January 31, 2020 from 050.197.099.173
Copyright © 2020 American Society of Clinical Oncology. All rights reserved.

19. Jeronimo J, Bansil P, Lim J, et al: A multicountry evaluation of CareHPV testing, visual inspection with acetic acid, and Papanicolaou testing for the detection of cervical cancer. Int J Gynecol Cancer 24:576-585, 2014

20. National Institutes of Health: NCT01231945: Low-Cost Molecular Cervical Cancer Screening Study. 2010 https://clinicaltrials.gov/show/NCT01231945

21. PATH: PATH announces new cervical cancer test for low-resource countries: CareHPV, a rapid, highly sensitive DNA test, could revolutionize cervical cancer screening where it is needed most. Press release: Seattle, November 28, 2012. http://www.path.org/news/press-room/147/

22. Schweizer J, Berard-Bergery M, Bisht A, et al: E6 Based Rapid Diagnostic Test for Cervical Pre-Cancer and Cancer. 2011. http://arborvita.com/news_press/media/AVantageHPVE6Dx-1.pdf

23. Qiao YL, Sellors JW, Eder PS, et al: A new HPV-DNA test for cervical-cancer screening in developing regions: A cross-sectional study of clinical accuracy in rural China. Lancet Oncol 9:929-936, 2008

24. Crosby RA, Hagensee ME, Vanderpool R, et al: Community-based screening for cervical cancer: A feasibility study of rural Appalachian women. Sex Transm Dis 42:607-611, 2015

25. Valdez M, Jeronimo J, Bansil P, et al: Effectiveness of novel, lower cost molecular human papillomavirus-based tests for cervical cancer screening in rural china. Int J Cancer 138:1453-1461, 2016

26. Penaranda E, Molokwu J, Flores S, et al: Women's attitudes toward cervicovaginal self-sampling for high-risk HPV infection on the US-Mexico border. J Low Genit Tract Dis 19:323-328, 2015

27. Adamson PC, Huchko MJ, Moss AM, et al: Acceptability and accuracy of cervical cancer screening using a self-collected tampon for HPV messenger-RNA testing among HIV-infected women in South Africa. PLoS One 10: e0137299, 2015

28. Verdoodt F, Jentschke M, Hillemanns P, et al: Reaching women who do not participate in the regular cervical cancer screening programme by offering self-sampling kits: A systematic review and meta-analysis of randomised trials. Eur J Cancer 51:2375-2385, 2015

29. Moses E, Pedersen HN, Mitchell SM, et al: Uptake of community-based, self-collected HPV testing vs. visual inspection with acetic acid for cervical cancer screening in Kampala, Uganda: Preliminary results of a randomised controlled trial. Trop Med Int Health 20:1355-1367, 2015

30. Rositch AF, Gatuguta A, Choi RY, et al: Knowledge and acceptability of pap smears, self-sampling and HPV vaccination among adult women in Kenya. PLoS One 7:e40766, 2012

31. Bansil P, Wittet S, Lim JL, et al: Acceptability of self-collection sampling for HPV-DNA testing in low-resource settings: A mixed methods approach. BMC Public Health 14:596, 2014

32. Vallès X, Murga GB, Hernández G, et al: High prevalence of human papillomavirus infection in the female population of Guatemala. Int J Cancer 125:1161-1167, 2009

33. Petrocy A, Katz ML: Cervical cancer and HPV: Knowledge, attitudes, beliefs, and behaviors among women living in Guatemala. J Health Care Poor Underserved 25:624-636, 2014

34. Department of Health and Human Services, Centers for Disease Control and Prevention: Prevention of Genital HPV Infection and Sequelae: Report of an External Consultants' Meeting. 1999. http://www.cdc.gov/std/hpv/HPVSupplement99.pdf

35. Eunice Kennedy Shriver National Institute of Child Health and Human Development, University of North Carolina Carolina Population Center: UNC Family Health Study Survey. 2010. http://www.unc.edu/~ntbrewer/surveys/HPV_2006_person.pdf

36. Villa LL: Cervical cancer in Latin America and the Caribbean: The problem and the way to solutions. Cancer Epidemiol Biomarkers Prev 21:1409-1413, 2012

37. Bychkovsky BL, Ferreyra ME, Strasser-Weippl K, et al: Cervical cancer control in Latin America: A call to action. Cancer 122:502-514, 2016

38. Jeronimo J, Holme F, Slavkovsky R, et al: Implementation of HPV testing in Latin America. J Clin Virol 76:S69-S73, 2016

39. Rosenbaum AJ, Gage JC, Alfaro KM, et al: Acceptability of self-collected versus provider-collected sampling for HPV DNA testing among women in rural El Salvador. Int J Gynaecol Obstet 126:156-160, 2014

40. Messmer SE. A Pilot Study on Women's Health Education in Rural Guatemala: Impact on Beliefs and Behaviors [doctoral dissertation]. Harvard Medical School, Boston, MA, 2014

41. Organización Mundial de la Salud: Manual de vigilancia STEPS de la OMS: El método STEPwise de la OMS para la vigilancia de los factores de riesgo de las enfermedades crónicas. 2006. http://goo.gl/06Th0Y

42. Hesselink AT, Sahli R, Berkhof J, et al: Clinical validation of Anyplex™ II HPV HR Detection according to the guidelines for HPV test requirements for cervical cancer screening. J Clin Virol 76:36-39, 2016

43. Bruni L, Diaz M, Castellsagué X, et al: Cervical human papillomavirus prevalence in 5 continents: Meta-analysis of 1 million women with normal cytological findings. J Infect Dis 202:1789-1799, 2010

44. Abrahams-Gessel S, Denman CA, Montano CM, et al: The training and fieldwork experiences of community health workers conducting population-based, noninvasive screening for CVD in LMIC. Glob Heart 10:45-54, 2015

Downloaded from ascopubs.org by 50.197.99.173 on January 31, 2020 from 050.197.099.173
Copyright © 2020 American Society of Clinical Oncology. All rights reserved.

45. Nagata JM, Barg FK, Valeggia CR, et al: Coca-colonization and hybridization of diets among the Tz'utujil Maya. Ecol Food Nutr 50:297-318, 2011

46. Nagata JM, Valeggia CR, Barg FK, et al: Body mass index, socio-economic status and socio-behavioral practices among Tz'utujil Maya women. Econ Hum Biol 7:96-106, 2009

47. Mitchell S, Ogilvie G, Steinberg M, et al: Assessing women's willingness to collect their own cervical samples for HPV testing as part of the ASPIRE cervical cancer screening project in Uganda. Int J Gynaecol Obstet 114:111-115, 2011

48. ICO Information Centre on HPV and Cancer: Guatemala Human Papillomavirus and Related Cancers, Fact Sheet 2016. http://www.hpvcentre.net/statistics/reports/GTM_FS.pdf

49. Pan American Health Organization (PAHO): Cervical Cancer Prevention and Control Programs: A Rapid Assessment in 12 Countries of Latin America. Washington, DC, PAHO, 2010

Downloaded from ascopubs.org by 50.197.99.173 on January 31, 2020 from 050.197.099.173
Copyright © 2020 American Society of Clinical Oncology. All rights reserved.
